# Extraction of active pharmaceutical ingredients from simulated spent activated carbonaceous adsorbents

**DOI:** 10.1007/s11356-020-08822-0

**Published:** 2020-04-30

**Authors:** Pierre Oesterle, Richard H Lindberg, Jerker Fick, Stina Jansson

**Affiliations:** Department of Chemistry, Umeå University, SE 90187 Umeå, Sweden

**Keywords:** Activated biochar, Activated carbon, Regeneration, Pharmaceuticals, API, Adsorption, Micropollutants

## Abstract

Activated carbon (AC) and activated biochar (ABC) are widely used as sorbents for micropollutant removal during water and wastewater treatment. Spent adsorbents can be treated in several ways, e.g., by incineration, disposal in landfills, or reactivation. Regeneration is an attractive and potentially more economically viable alternative to modern post-treatment practices. Current strategies for assessing the performance of regeneration techniques often involve only repeated adsorption and regeneration cycles, and rarely involve direct measurements of micropollutants remaining on the adsorbent after regeneration. However, the use of regenerated adsorbents containing such residual micropollutants could present an environmental risk. In this study, the extraction of eight active pharmaceutical ingredients (APIs) commonly found in treated effluents was evaluated using 10 solvents and sorption onto three different carbon materials. An optimized extraction method was developed involving ultrasonication in 1:1 methanol:dichloromethane with 5% formic acid. This method achieved recoveries of 60 to 99% per API for an API concentration of 2 μg/g char and 27 to 129% per API for an API concentration of 1 mg/g char. Experiments using a mixture of 82 common APIs revealed that the optimized protocol achieved extraction recoveries above 70% for 29 of these APIs. These results show that the new extraction method could be a useful tool for assessing the regenerative properties of different carbon sorbents.

**Electronic supplementary material:**

The online version of this article (10.1007/s11356-020-08822-0) contains supplementary material, which is available to authorized users.

## Introduction

The per capita consumption of pharmaceuticals (including antibiotics, antidepressants, and painkillers) is increasing around the world (Bernhardt et al. 2017; Klein et al. 2018). When ingested, some active pharmaceutical ingredients (APIs) are partially metabolized, while others remain unchanged. After leaving the body, these degradation products and the non-degraded APIs end up in wastewater treatment plants (WWTPs). Most WWTPs were originally designed to reduce nutrient pollution and are therefore ineffective at removing micropollutants. Consequently, these substances are commonly released into downstream ecosystems (Lindberg et al. 2014; Melvin and Leusch 2016; Östman et al. 2018), where they have been shown to have many adverse environmental and ecological effects, including causing changes in the behavior of fish populations (Brodin et al. 2013) and promoting the transmission of antibacterial resistance genes to pathogenic bacteria (Baquero et al. 2008). To prevent these undesirable outcomes, many WWTPs have implemented tertiary treatment procedures designed to degrade (e.g., ozonation) or capture (using materials such as adsorbents) these contaminants before they enter the environment.

A wide range of different materials are currently used as adsorbents in wastewater treatment. The choice of material depends on the water’s contamination profile, the concentrations of contaminants, and the required removal capacity (Mohan et al. 2014). Activated carbon (AC) adsorbents are typically very efficient, but they are also expensive and have high environmental impacts due to their limited lifetime, fossil feedstock origin, and energy-intensive production, as well as the consumption of chemicals during their activation, and/or the need to transport them over long distances (Thompson et al. 2016). Biochars are carbon-rich porous materials formed by pyrolysis of different types of biomass. They resemble commercial ACs, particularly after activation, and can therefore be used as adsorbent materials. The removal of organic micropollutants such as pharmaceuticals and biocides from simulated wastewater using biochars made from materials such as pyrolysed crop residues has been demonstrated (Mohan et al. 2014; Weidemann et al. 2018). Although ACs and activated biochars (ABCs) could be used in wastewater treatment plants to adsorb APIs, it is important to note that sorption of contaminants onto a solid material may only displace the problem; environmental harm may still occur during subsequent steps when the used adsorbents are disposed of. Incineration is the most common disposal method for spent carbon adsorbents, and the most cost-effective way of using low-cost biochars from abundant local feedstocks will probably be to use them only once before disposal (Ahmad et al. 2014; Mohan et al. 2014). Nevertheless, there are alternatives, including physical, chemical, or biological regeneration of the used adsorbent (Salvador et al. 2015a; El Gamal et al. 2018; Salvador et al. 2015b).

While some studies have identified potentially safe and sustainable regeneration technologies for carbonaceous adsorbents, further research is needed to confirm that these techniques allow char to be reused without altering its porous structure, causing substantial mass loss, or leading to accumulation of micropollutants on the adsorbent over time. Some methods, such as electrochemical or hydrothermal regeneration (Sühnholz et al. 2018) may cause partial fragmentation of the contaminants followed by their re-adsorption onto the activated adsorbent. Contaminants may thus remain on the surface of the regenerated adsorbent and be subject to multi-layered adsorption. The quantities of contaminants remaining on the regenerated adsorbent can be determined by extracting the APIs using extraction techniques such as microwave-assisted extraction, pressurized liquid extraction, or supercritical fluid extraction (Vom Eyser et al. 2015; Zuloaga et al. 2012). However, these techniques often require expensive equipment. Ultrasonic bath extraction offers an inexpensive and fast alternative (Martínez-Parreño et al. 2008).

The aim of this study was to develop, optimize, and evaluate a fast and operationally simple multi-residue extraction method. To this end, selected contaminants were adsorbed on granular AC and then extracted. The extracts were analyzed by liquid chromatography coupled with tandem mass spectrometry (LC-MS/MS). This method was tested on one granular AC commonly used in drinking water treatment facilities and two powdered ABCs, and optimized using a set of 8 APIs commonly prescribed around the world, which were applied to the tested adsorbents at two different concentrations. To assess the optimized method’s performance for a larger set of APIs, a second set of experiments was performed using a mixture of 82 APIs to better represent the diverse contaminant profile commonly found in treated WWTP effluents.

## Materials and methods

### Standard, internal standards, and solvents

Stock solutions of 8 APIs for the initial optimization experiments and 82 APIs for the large-scale screening were prepared in methanol (HPLC grade, Fisher Chemical). The APIs used in each set of experiments are listed in the Supplementary information (Table S1). Deionized (DI) water was used to dilute the APIs, in the adsorption experiments, and during the analysis and quantification of APIs using LC-MS/MS. Extraction was performed using high purity (> 99%) dichloromethane, acetonitrile, methanol, and toluene (all from Fisher Chemical), and formic acid (FA) of 98–100% purity (Merck).

### Adsorbents and preconditioning

Biochars were produced and activated at the UK Biochar Research Centre (UKBRC) at the University of Edinburgh, UK. Softwood pellet and wheat straw pellet biochars generated by pyrolysis at 700 °C were subsequently activated with a flow of CO_2_ at 800 °C for 1 h. The activated straw- and wood-derived biochars are referred to as ABC-S and ABC-W, respectively. These biochars were selected because they are well characterized and available in large quantities. Furthermore, they are among the standardized reference biochar materials developed by the UK Biochar Research Centre (UKBRC) at the University of Edinburgh (the so-called Edinburgh Standard Biochar set) which facilitates replication of research results, comparison of findings between studies (Mašek et al. 2018a). The biochars differ in their total carbon; ash content; pH; electrical conductivity; total P, K, and N; and surface area (Mašek et al. 2018b). Aquasorb 2000 (Jacobi) was chosen as a reference AC material because it is commonly used for water treatment and therefore widely available. ABC-W and ABC-S were ground and sieved to obtain a homogeneous particle size of 0.125–0.5 mm. After sieving, they were acid-washed with 0.1 M HCl to remove most of the inorganic salts on the surface and thereby reduce sample variability.

Prior to extraction, the AC and ABCs were loaded with 15 mL of API solution at a concentration of 1 μg/L (300 ng of API per g of char). To assess the performance of the optimized extraction method, the AC and ABCs were also loaded with 8 mL of API solution at a concentration of 1 mg/L (160 μg of API per g of char). To maximize sorption, the adsorbents were left in contact with the solution in falcon tubes for 24 h under continuous rotation using an in-house designed tube rotator and at ambient temperature. In addition to the triplicate extraction tests, an extraction blank containing only the APIs in solution was analyzed to account for potential degradation occurring over 24 h. To explore potential matrix effects due to the degradation of the AC/ABCs during ultrasonication, blanks containing only AC/ABCs and DI water were analyzed. The falcon tubes were subsequently centrifuged at 4700 rpm for 10 min at ambient temperature, and the supernatant containing the remaining non-sorbed APIs was analyzed by LC-MS/MS to correct the extraction results. The remaining water in the AC and ABC materials was then carefully removed to minimize the volume of the water phase in the subsequent ultrasound-assisted extraction.

### Ultrasonic extraction

Preliminary experiments were conducted to identify conditions enabling efficient API desorption from the reference AC material. After optimization on AC, the method was tested on the two ABC adsorbents. A previously reported ultrasonication method originally developed for use with soil, sediments, and carbon nanotubes was used to desorb the APIs from the loaded AC and ABCs (Martín et al. 2010; Mason et al. 2004; Okuda et al. 2009; Wang et al. 2017). Sonication of a liquid or suspension generates air bubbles that expand and then collapse. Their collapse creates shockwaves that cause the AC/ABC particles to fragment, increasing the surface area exposed to the extraction solvents (Mason et al. 2004; Yin et al. 2017). Because the solvent’s polarity and acidity influence contaminant desorption (Martínez-Carballo et al. 2007; Reguyal et al. 2017; Martínez-Parreño et al. 2008), 10 extraction solvents were tested, including the nonpolar solvents toluene and dichloromethane (DCM), the polar protic solvent methanol (MeOH), the dipolar aprotic solvent acetonitrile (ACN), and 1:1 binary mixtures of these solvents (5 mL per extraction). The biochars were sonicated for 20 min at 20 °C and centrifuged for 10 min at 4700 rpm, after which the supernatant was transferred to a 16 mL glass tube and evaporated under an air stream at 32 °C. The APIs were resolubilised with 10 mL of DI water spiked with 10 μL of FA and 26 μL of an internal standard mix containing deuterated ciprofloxacin, sulfamethoxazole, carbamazepine, tamoxifen, promezathine, amitryptiline, oxazepam, risperidone, tramadol, trimethoprim, paracetamol, codeine, flecainide, diclofenac, clotrimazole, and fluconazole. The solution was then filtered through a particle filter with a 0.45 μm pore size to remove AC/ABC particles, and the filtrate was analyzed by an online solid phase extraction and LC-MS/MS. Each experiment was performed in triplicate, and the APIs were quantified relative to the corresponding deuterated internal standard. The API’s sorption was evaluated by accounting for the differences between the initial concentration of APIs, the concentration in the DI water after the 24-h adsorption, and the results obtained after extraction.

The method was then optimized by co-varying the extraction time (5–20 min), number of extraction cycles (*n* = 1–3), and the amount of FA added to the extraction solvents (0–5%). To assess the versatility of the extraction method, the conditions yielding the highest recoveries were tested in an expanded trial including two ABC adsorbents (ABC-S and ABC-W) in addition to the previously evaluated AC adsorbent and with two levels of sorbent loading (achieved by using mixed API solutions with concentrations of 2 μg/L and 1 mg/L). The experiments were also performed using mixed API solutions containing an expanded set of 82 APIs at a concentration of 1 μg/L (300 ng of API per g of char) to identify those that could be extracted with recoveries above 70%.

### Quantification with LC-MS-MS

LC-MS/MS analysis was performed using the online solid phase extraction and liquid chromatography tandem mass spectrometry, as described in detail elsewhere (Lindberg et al. 2014). The APIs were enriched online using an OASIS HLB (20 mm × 2.1 mm i.d., 15 mm particle size, Waters, Milford, MA, USA). API separation was performed using a Hypersil GOLD aQ C18 Polar encapped guard column (20 mm × 2.1 mm i.d.m, 5 μm particle size, Thermo Fisher Scientific, San Jose, CA, USA) and an LC column (50 mm × 2.1 mm i.d., 5 μm particle size) with a mobile phase gradient of water and acetonitrile (both containing 0.1% FA) varying from 0 to 100% acetonitrile. The APIs were then analyzed using a TSQ Quantum Ultra EMR triple quadrupolemass spectrometer (Thermo Fisher Scientific, San Jose, CA, USA) and quantified against internal standards. The limits of quantification for each tested API are listed in Table S1 (Supplementary information).

## Results and discussion

### Solvent selection and optimization of an API’s training set

While extraction solvents are usually selected-based solely on the polarity of the target contaminants, an alternative approach is to mix miscible polar and nonpolar solvents to enable the desorption of a wider range of APIs. This approach was adopted in a recent study that used an ultrasonication-assisted method to extract PAHs from biochars (Chen et al. 2015).

As noted above, the extraction method for the 8 selected APIs was initially tested on AC. In this first solvent assessment, only one extraction cycle was performed, using an extraction time of 10 min. To facilitate interpretation, the observed recoveries (Table 1) are shown relative to the best extraction recovery (RER) for the relevant API, so the highest recoveries obtained are set to 100%. It should be noted that this does not mean that the API was completely extracted from the char; instead, a value of 100% means that the specified combination of solvents yielded a higher recovery than any other tested combination. The numbers in parentheses show the absolute recoveries achieved for each solvent and contaminant.

**Table 1:** Relative extraction recoveries (RER) (%) of 8 APIs loaded on AC

			Solvent										
API	Log *P*	pKa	Toluene	DCM	MeOH	ACN	Tol/MeOH	Tol/DCM	Tol/ACN	DCM/MeOH	DCM/ACN	MeOH/ACN	Max Absolute Recovery (%)
Fluconazole	0.4	1.72	8 (7)	33 (32)	20 (20)	31 (30)	99 (96)	26 (26)	33 (32)	100 (97)	44 (42)	36 (35)	97
Trimethoprim	0.9	7.12	10 (7)	23 (16)	34 (24)	46 (32)	100 (70)	31 (22)	48 (33)	100 (70)	44 (31)	46 (32)	70
Carbamazepin	2.3	15.96	31 (31)	67 (66)	18 (17)	37 (36)	100 (98)	77 (76)	65 (64)	92 (91)	63 (62)	28 (28)	98
Tramadol	2.4	9.23/13.08	18 (15)	48 (40)	38 (31)	44 (37)	81 (68)	52 (43)	58 (49)	100 (83)	58 (48)	49 (41)	83
Oxazepam	2.8	1.55/10.9	14 (8)	24 (13)	29 (17)	29 (16)	100 (57)	31 (18)	32 (18)	98 (56)	31 (18)	40 (23)	57
Flecainide	4.6	9.3	29 (23)	40 (32)	63 (50)	60 (48)	91 (73)	53 (42)	58 (47)	100 (80)	55 (44)	66 (53)	80
Amitriptyline	4.9	9.76	42 (28)	56 (38)	64 (44)	64 (44)	98 (67)	75 (51)	65 (44)	100 (68)	63 (43)	66 (45)	68
Clotrimazole	5	4.1	36 (34)	58 (55)	71 (67)	68 (65)	88 (83)	68 (64)	60 (56)	100 (94)	67 (63)	69 (66)	94

Recoveries achieved with specific solvents or solvent mixtures are given as percentages relative to the best extraction recovery for the API in question, which is shown in the Max Absolute Recovery column. *DCM*, dichloromethane; *MeOH*, Methanol; *ACN*, acetonitrile; *Tol*, toluene. Log *P* and pK_*a*_ values were obtained using PubChem

Mixed solvent systems generally yielded higher RER values than single solvents (Table 1). The results were compared with the compound’s log *P* value, i.e., the partition coefficient between organic and aqueous phase. Substances with log *P* < 3 will more favorably partition into the aqueous phase, and thereby becomes more bioavailable. Substances with log *P* > 3 on the other hand are more lipophilic. Toluene consistently had the lowest RER and is, like DCM, a nonpolar solvent immiscible in water. The AC was extracted on wet basis to avoid degradation of contaminants during drying. Therefore, it is likely that water molecules were still present in the pores of the biochars during the extraction, which would prevent nonpolar solvents from accessing the solvated APIs. Compared with toluene, DCM yielded RERs that were up to three times higher for more hydrophilic compounds (log *P* < 3) and 1.5 times higher for more hydrophobic compounds (log *P* > 3). This is probably because DCM is more polar than toluene.

For hydrophilic APIs, the RERs achieved with MeOH were generally similar to those achieved with DCM; the lone exception was carbamazepine, for which DCM had a substantially higher RER (67 vs 18%). Conversely, for hydrophobic APIs, the RERs achieved with DCM were equal to or less than those achieved with ACN and MeOH. Toluene and DCM are immiscible in water, which may affect the way they interact with adsorbed compounds during solid-liquid extraction; these results suggest that hydrophobic APIs preferentially remained sorbed on the surface of the char (possibly in its hydrated pores) during wet extractions. Because MeOH and ACN are polar and miscible with water, they yielded higher RERs than toluene and DCM for most of the hydrophilic APIs. Their RERs were also generally comparable to those of DCM for hydrophobic APIs.

Extractions were also performed using binary solvent mixtures. Mixtures of toluene with DCM or ACN yielded RERs similar to those observed with DCM or ACN alone. Similarly, the MeOH/ACN mixture did not perform appreciably better than its individual components. The Tol/DCM, Tol/ACN, and MeOH/ACN combinations did not improve the RER because both components of these mixtures have similar properties and are thus good solvents for similar compound types. The highest absolute extraction recoveries (ranging from 57 to 98%) were used with the Tol/MeOH and DCM/MeOH mixtures, which have one polar and non-polar component. The best recoveries were achieved with DCM/MeOH (1:1), so this solvent combination was used in all subsequent experiments.

To further optimize the ultrasonication method and improve the recovery of the selected APIs, three factors were varied: the number of extraction cycles (*n* = 1–3), the duration of extraction (5–20 min), and the quantity of FA added to the extraction solvent (0–5%). Because methanol is amphoteric (i.e., capable of acting as either a Brönsted base or a Brönsted acid) while DCM is neutral, adding FA could improve the extraction of compounds whose solubility is pH-dependent. Seventeen experiments were conducted based on a D-optimal design but obtained no statistically significant results. However, adding FA increased the extraction efficiencies of trimethoprim, oxazepam, and amitriptyline by 10–20% for single-cycle extractions with durations of 5 min. Calculations of the solubilities of these compounds (logS) as a function of pH (Chemaxon 2019) suggested that trimethoprim and amitriptyline are more soluble under acidic conditions (pH < 5), which may partly explain why adding FA increased their efficiency of extraction. However, oxazepam’s calculated solubility is pH-independent between pH 1 and pH 12, so the extraction recovery could be influenced by the presence of other analytes. Based on the results from the experimental design, the protocol chosen for the expanded extraction tests using three adsorbents, and eight APIs involved two 20-min extraction cycles with 5% FA and DCM:MeOH 1:1.

### Evaluation of the optimized method using additional adsorbents at low and high concentrations

The performance of the optimized method was assessed using two additional adsorbents with different physical and chemical properties (ABC-S and ABC-W), at low and high API concentrations.

Guidelines published by the International Council for Harmonization of Technical Requirements for Pharmaceuticals for Human Use (ICH) state that extraction methods for analytical procedures should achieve recovery rates of 70–130% (ICH-Harmonised-tripartite-guideline 2005). At low concentrations (2 μg/g of char), the optimized extraction method achieved satisfactory recovery rates (78–99%) for AC (Table 2). The recoveries achieved for ABC-S-L and ABC-W-L ranged from 60 to 82% and 87 to 96%, respectively. ABC-S-L consistently afforded lower recoveries than ABC-W-L and AC, possibly because of differences in adsorption and pore structure between ABCs and AC. Therefore, at low API loadings, the optimized extraction method only complies with the ICH guidelines for ABC-W and AC.

**Table 2:** Extraction recoveries (%) of 8 APIs using the optimized extraction method for three adsorbent materials at low (2 μg of API per gram of char) and high (1 mg of API per gram of char) API concentrations

Recoveries (%)	Adsorbent					
API	AC-L	AC-H	ABC-W-L	ABC-W-H	ABC-S-L	ABC-S-H
Amitriptyline	81 ± 6	92 ± 6	87 ± 3	129 ± 26	65 ± 4	129 ± 19
Carbamazepine	87 ± 5	93 ± 5	91 ± 4	85 ± 9	76 ± 5	101 ± 1
Clotrimazole	98 ± 4	27 ± 3	96 ± 2	52 ± 38	80 ± 2	85 ± 14
Flecainide	89 ± 6	99 ± 2	96 ± 3	86 ± 6	67 ± 3	90 ± 3
Fluconazole	99 ± 5	99 ± 3	97 ± 3	88 ± 7	82 ± 3	96 ± 1
Oxazepam	78 ± 4	74 ± 3	91 ± 2	61 ± 13	69 ± 4	74 ± 3
Tramadol	88 ± 6	95 ± 1	92 ± 2	90 ± 4	60 ± 3	83 ± 1
Trimethoprim	80 ± 5	92 ± 1	79 ± 4	74 ± 6	48 ± 6	89 ± 2

*ABC-W*, softwood biochar activated at 800 °C; *ABC-S*, wheat straw biochar activated at 800 °C; and *AC*, activated carbon. The suffixes -L and -H denote chars loaded with low and high API concentrations, respectively, prior to extraction

At the higher API loading (1 mg/g of char), acceptable recoveries were achieved for all APIs and adsorbents other than for clotrimazole from ABC-W-H and AC-H and oxazepam from ABC-W-H. Clotrimazole has limited solubility in water (0.49 mg/L), which may explain its poor recoveries from ABC-W-H and AC-H. It was not possible to explain why the recovery of oxazepam from ABC-W-H was lower than that from ABC-W-L but increasing the number of extraction cycles could potentially improve the recovery of this API. In the case of amitriptyline, the recoveries achieved at the high API loading were as high as 129%, and were 42 to 64% higher than those achieved at the low API loading. However, the recoveries were still in the 70–130% range at the low loading.

Many inherent material properties influence the adsorption of organic compounds on ACs/ABCs, including the surface area, pore size, pore volume, zeta potential, isoelectric point, ash content, and surface functional groups (Kah et al. 2017). These properties correlate strongly with the composition of the feedstock and the pyrolysis/activation conditions, and will primarily affect the mechanism by which a substance is sorbed onto the surface of an AC/ABC (Tong et al. 2019). Extraction recoveries also depend on the adsorbate’s properties. The APIs used in these experiments include acidic, basic, and amphoteric compounds, which complicates prediction of their efficiencies of extraction. The differences in recovery between AC, ABC-W, and ABC-S are probably related to differences in adsorbent-adsorbate interactions.

### Evaluation of the optimized method using an extended test set

Due to the observed differences in extraction efficiencies within the small set of APIs (*n* = 8), experiments were performed with an expanded API set to further evaluate the new protocol’s ability to induce desorption from the three activated adsorbent materials. A mix of 82 APIs at concentrations of approximately 1 μg/L was therefore tested, with the aim of achieving 80–100% adsorption of the APIs onto the surface of the tested sorbent materials.

The selected APIs were grouped according to the Anatomical Therapeutic Chemical (ATC) classification system (Table S3, Supplementary information). This system groups APIs based on chemical similarity and mode of action, and features five levels of classification (Table 3 and Table S3). The 82 APIs used in the experiment were assigned to 54 different subgroups, 12 of which contained two or more APIs. All APIs belonging to the same subgroup exhibited similar extraction recovery profiles. The 12 subgroups containing multiple APIs were beta-blocking agents (selective), angiotensin II, HMG CoA reductase inhibitors, tetracyclines, macrolides, fluoroquinolones, tertiary amines, phenothiazines with piperazine structure, thioxanthene derivatives, non-selective monoamine re-uptake inhibitors, selective serotonin re-uptake inhibitors, and aminoalkyl ethers, and other (Table 3).

**Table 3:**
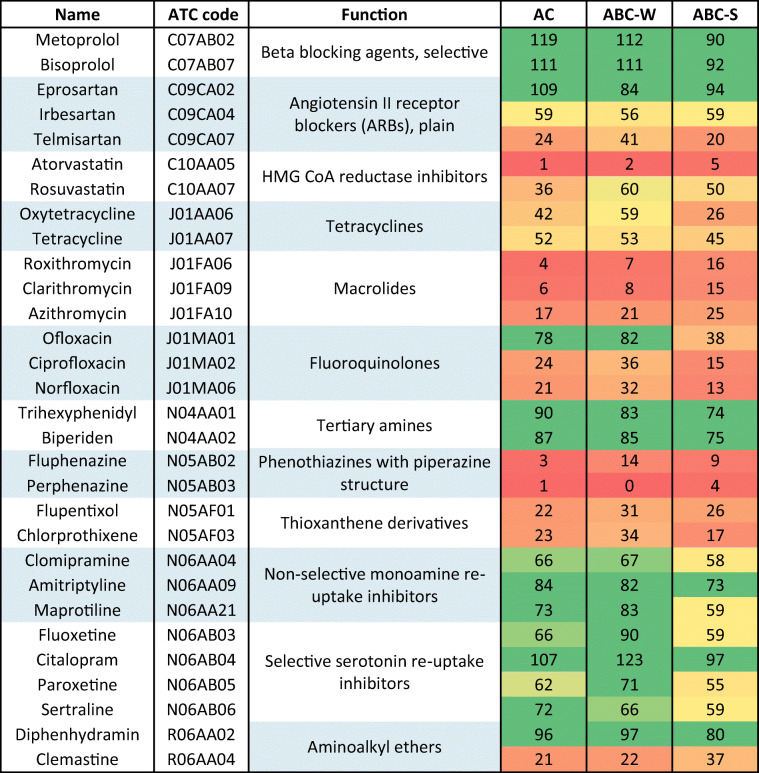
Extraction recoveries of specific pharmaceutical groups from the three activated carbon adsorbents

The colors in the extraction recovery columns indicate the efficiency of the corresponding extraction method, < 50% recoveries are shown in red, < 70% recoveries are shown in yellow, and > 70% are shown in green

APIs belonging to three of the 12 groups (macrolides, phenothiazines with piperazine structures, and thioxanthene derivatives) exhibited consistently poor extraction recoveries (< 40%). Macrolides are hydrophobic compounds having a macrocyclic lactone ring with isolated or conjugated double-bonds that is attached to one or more amino-sugars, and are unstable under acidic conditions. Therefore, the use of FA in the extraction solvent may have degraded these APIs preventing their detection by mass spectrometry (Berrada et al. 2010). Fluphenazine and perphenazine, which are phenothiazines with piperazine structures, are structurally similar; they differ only in that they have different halogen substituents on their phenothiazine groups (chlorine for perphenazine and three fluorine substituents for fluphenazine). Flupentixol and chlorprothixene are thioxanthene derivatives, whose structures resemble those of the phenothiazines—both compound classes have a xanthene ring system in which the oxygen center is substituted by sulfur (thioxanthene) or nitrogen (phenothiazines). The extraction efficiencies of these APIs could potentially be improved by using EDTA as a chelating agent, as demonstrated in a study on the extraction of xanthene dyes from soils (Alcantara-Licudine et al. 1997). Tetracyclines, which have partially conjugated four-ring structures with a carboxamide functional group, exhibited poor to average (26–59%) extraction recoveries. Although these compounds are amphoteric (and thus soluble in both polar and nonpolar solvents), they can form strong complexes with multivalent cations such as Fe^2+^ (Fedeniuk and Shand 1998). Typical procedures for tetracycline extraction therefore involve the use of a chelating agent such as citric or oxalic acid to prevent them from binding to cations on the surface of the AC/ABC (Fedeniuk and Shand 1998). In this study, FA was used instead to avoid salt precipitation during LC-MS/MS analysis.

**Fig. 1:**
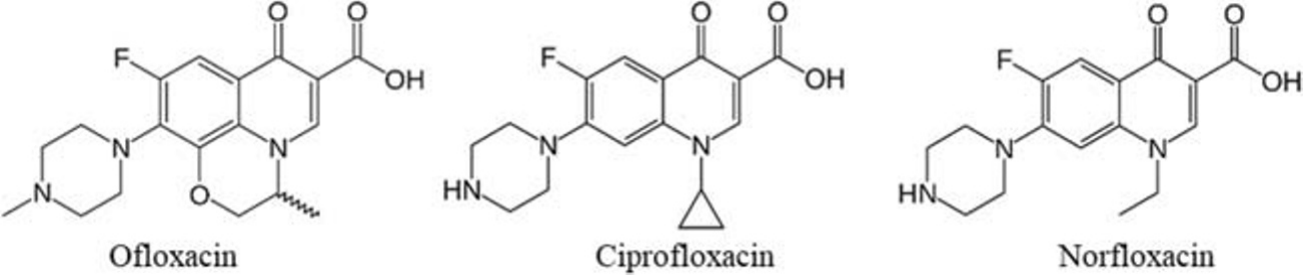
Chemical structures of the fluoroquinolones included in the study

The extraction recoveries of the three fluoroquinolones included in the experiment also show that minor differences in chemistry can affect the adsorption and extraction processes (Fig. 1). Ciprofloxacin and norfloxacin have almost identical chemical structures, differing only at one alkyl chain at one of their tertiary amines, and they also have similar extraction recoveries. Ofloxacin differs marginally from ciprofloxacin and norfloxacin in that it lacks a secondary amine and has a rigid cyclic structure instead of an alkyl chain at the tertiary amine. These minor differences significantly increase its extraction recoveries, implying that the adsorption of fluoroquinolones is highly site-specific and dependent on interactions between functional groups.

For the 6 other groups of APIs that exhibited average or good extraction recoveries, all three carbon adsorbents achieved similar extraction performances. This makes the newly developed extraction method suitable for some compounds in the following groups: beta blocking agents, tertiary amines, non-selective monoamine reuptake inhibitors, and selective serotonin reuptake inhibitors. It was not possible to test every API belonging to each group, so further group-specific studies are needed.

**Fig. 2:**
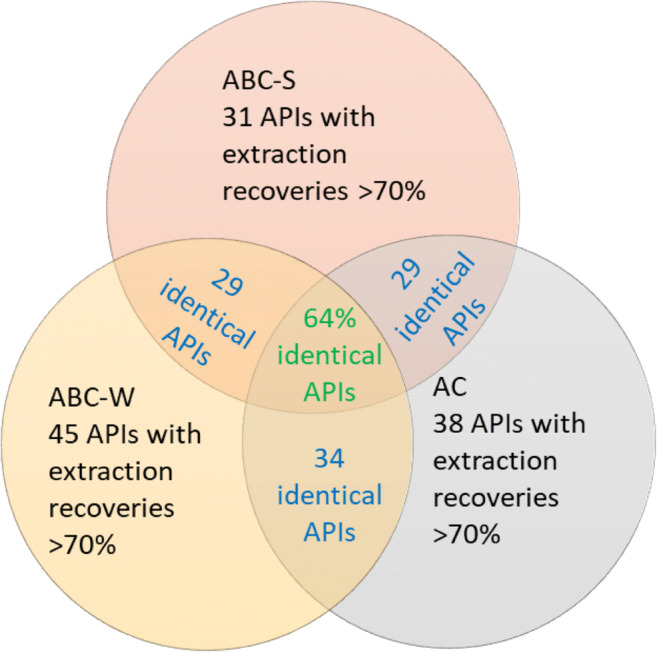
Venn diagram showing the overlaps in extraction recovery for the three carbon adsorbents and 82 APIs. *API*, active pharmaceutical ingredient; *AC*, activated carbon; *ABC-W*, activated biochar derived from wood; *ABC-S*, activated biochar derived from wheat straw

Different adsorbents have different recovery profiles because their different physicochemical properties affect the binding mechanisms of the APIs (Tong et al. 2019). Most of the APIs considered here had higher recoveries from AC and ABC-W than from ABC-S. To more clearly visualize the recoveries achieved with each activated adsorbent, a Venn diagram was created showing the numbers of APIs for which an extraction recovery of at least 70% was obtained (see Fig. 2).

Of the 82 APIs included in the study, between 31 and 45 had recoveries above 70% (based on the adsorbent’s initial loading) for at least one of the three tested adsorbents. A total of 29 APIs had absolute recoveries of at least 70% for all three adsorbents, corresponding to an overlap of 64% in the sets of APIs extracted efficiently by AC, ABC-W, and ABC-S. The extraction method developed in this work is thus applicable to the extraction of 29 APIs from at least three different adsorbents.

**Fig. 3:**
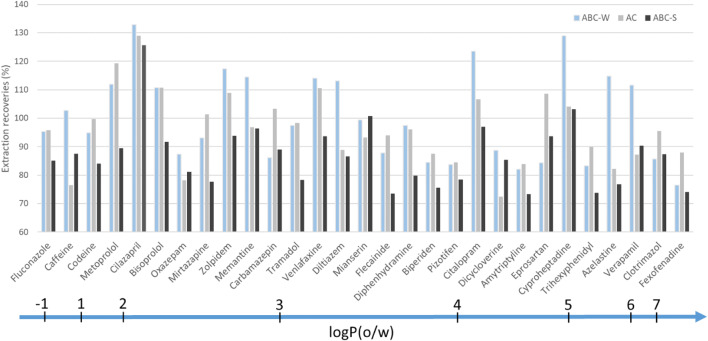
Extraction recoveries (%) of 29 APIs with the optimized extraction method

Figure 3 shows the APIs with high (> 70%) extraction recoveries sorted by the partition coefficient in neutral media between octanol and water (log *P*(*o*/*w*)), which ranged from − 1.1 to 7.3 for this set of compounds. Substances with log *P*(*o*/*w*) values ≤ 5 are highly water-soluble whereas substances such as fexofenadine, which has a log *P*(*o*/*w*) of 7.3, are more lipophilic (Bhal 2007). The extraction method presented here thus enables efficient extraction of a broad range of lipo- and hydrophilic APIs that are commonly found in treated wastewater effluents. These results are consistent with those of a previously published study on the extraction of natural plant products using a combination of methanol and dichloromethane (Sasidharan et al. 2010). Because the extraction protocol used in this work uses a mixture of hydrophobic and hydrophilic solvents, it may be that properties other than hydrophobicity drive the extraction of APIs from the surface of the carbon adsorbents. In our case, 64% of the tested compounds with recoveries above 70% for at least one adsorbent exhibited comparably good recoveries on all three adsorbents (Fig. 2), suggesting that API properties may be the main determinants of desorption efficiency for most of the APIs tested here. Further tests on more ACs and ABCs could reveal additional trends.

## Conclusion

An extraction method has been developed and validated that uses a combination of methanol, dichloromethane, and FA to remove APIs loaded on AC and ABC adsorbents. Reliable assessments of extraction efficiency are essential for evaluating the potential for regeneration of AC/ABC adsorbents. The DCM:MeOH solvent combination excelled at extracting a wide range of APIs because of the two solvents’ differing polarities, and the method’s performance was further improved by adding FA (5%), which increased extraction recovery by as much as 22% in the case of oxazepam. The initial extraction efficiency test indicated that the method presented here is a reliable way of assessing the extraction efficiency of AC/ABC adsorbents for a limited set of specific APIs spanning a wide range of hydrophilicities (logP between − 1.1 and 6) at both high and low API loadings (2 μg/g and 1 mg/g of API per char, respectively). For both loadings, the new extraction protocol achieved recoveries from AC and ABCs that were within the acceptable range for all eight initially tested APIs with the exception of clotrimazole. However, the results obtained suggested that the method’s performance was limited by the solubility of some of the APIs.

The extraction efficiencies of a larger set of 82 APIs were also analyzed, which varied between 0 and 130%. Twenty-nine APIs from this set of 82 were extracted successfully (i.e., had recoveries above 70%) from all three tested adsorbents. These results show that the method presented here could be useful for studying adsorbent regeneration and the interactions of AC and ABC adsorbents with APIs belonging to several different pharmaceutical groups. With further evaluation and verification, it could potentially serve as a simple and fast multi-residue extraction method for determination and verification of adsorbent regeneration efficiency, i.e., for ensuring that the residual concentrations of APIs on regenerated AC or ABC absorbents are below the LOQ, or that the residual APIs are irreversibly bound to the adsorbent and thus not susceptible to leaching**.**

## Electronic supplementary material

ESM 1 (DOCX 47 kb)
