# Supplementary material for: Extraction of active pharmaceutical ingredients from simulated spent activated carbonaceous adsorbents
Source: Environ Sci Pollut Res Int. 2020 Apr 30;27(20):25572–81. doi: 10.1007/s11356-020-08822-0 (PMC7329761; doi:10.1007/s11356-020-08822-0)
Supplement: Supplementary file 1 — (DOCX 47 kb) [file 11356_2020_8822_MOESM1_ESM.docx]

**Supporting information**

**Extraction of active pharmaceutical ingredients from simulated spent activated carbonaceous adsorbents**

Pierre Oesterle^a^; Richard H Lindberg^a^; Jerker Fick^a^; Stina Jansson^a^*

^a^ Department of Chemistry, Umeå University, SE 90187 Umeå, Sweden

*corresponding author, e-mail: stina.jansson@umu.se, phone: +46-(0)90-7867622, +46-(0)70-6567217

**Table S1.** The active pharmaceutical substances included in the study, with CAS numbers and limit of quantification (LOQ).

| Name | CAS | LOQ (ng/g) |
| --- | --- | --- |
| Alfuzosin | 81403-68-1 | 0.1 |
| Alprazolam | 28981-97-7 | 10 |
| Amytriptyline | 549-18-8 | 5 |
| Atorvastatin | 134523-00-5 | 50 |
| Atracurium | 64228-79-1 | 0.5 |
| Azelastine | 58581-89-8 | 5 |
| Azithromycin | 83905-01-5 | 5 |
| Beclometasone | 4419-39-0 | 10 |
| Biperiden | 1235-82-1 | 0.1 |
| Bisoprolol | 66722-45-0 | 0.1 |
| Bromocriptine | 25614-03-3 | 5 |
| Buprenorphine | 53152-21-9 | 10 |
| Bupropion | 31677-93-7 | 0.1 |
| Caffeine | 58-08-2 | 50 |
| Carbamazepin | 298-46-4 | 1 |
| Cilazapril | 92077-78-6 | 1 |
| Citalopram | 59729-33-8 | 5 |
| Clarithromycine | 81103-11-9 | 1 |
| Clemastine | 14976-57-9 | 0.5 |
| Clindamycine | 21462-39-5 | 1 |
| Clomipramine | 17321-77-6 | 0.5 |
| Clonazepam | 1622-61-3 | 5 |
| Clotrimazol | 23593-75-1 | 1 |
| Codeine | 1422-07-7 | 0.5 |
| Cyproheptadine | 969-33-5 | 5 |
| Desloratidin | 100643-71-8 | 0.5 |
| Dicycloverine | 77 - 19 - 0 | 5 |
| Dihydroergotamine | 6190-39-2 | 50 |
| Diltiazem | 42399-41-7 | 0.5 |
| Diphenhydramine | 147-24-0 | 0.05 |
| Dipyridamole | 58-32-2 | 1 |
| Donepezil | 120011-70-3 | 0.5 |
| Duloxetine | 116539-59-4 | 1 |
| Eprosartan | 144143-96-4 | 5 |
| Fexofenadine | 153439-40-8 | 5 |
| Finasteride | 98319-26-7 | 10 |
| Flecainide | 54143-55-4 | 0.1 |
| Fluconazole | 86386-73-4 | 0.5 |
| Flunitrazepam | 1622-62-4 | 10 |
| Fluoxetine | 54910-89-3 | 5 |
| Flupentixol | 30909-51-4 | 5 |
| Fluphenazine | 5002-47-1 | 10 |
| Flutamide | 13311-84-7 | 5 |
| Haloperidol | 52-86-8 | 0.1 |
| Hydroxyzine | 1244-76-4 | 0.5 |
| Irbesartan | 138402-11-6 | 0.5 |
| Loperamide | 34552-83-5 | 0.5 |
| Maprotiline | 10347-81-6 | 5 |
| Meclozine | 1104-22-9 | 5 |
| Metoprolol | 56392-17-7 | 5 |
| Mianserin | 21535-47-7 | 1 |
| Mirtazapine | 61337-67-5 | 10 |
| Naloxone | 465-65-6 | 1 |
| Nefazodone | 83366-66-9 | 0.5 |
| Ofloxacin | 82419-36-1 | 10 |
| Orphenadrine | 341-69-5 | 0.1 |
| Oxazepam | 604-75-1 | 5 |
| Oxytetracycline | 2058-46-0 | 50 |
| Paroxetine | 61869-08-7 | 10 |
| Pizotifen | 15574-96-6 | 0.5 |
| Propranolol | 525-66-6 | 50 |
| Ranitadine | 66357-35-5 | 5 |
| Repaglinide | 135062-02-1 | 0.5 |
| Risperidone | 106266-06-2 | 0.1 |
| Rosuvastatin | 147098-20-2 | 10 |
| Roxithromycine | 80214-83-1 | 50 |
| Sertraline | 79559-97-0 | 10 |
| Sotalol | 959-24-0 | 0.5 |
| Telmisartan | 144701-48-4 | 50 |
| Tetracycline | 64-75-5 | 50 |
| Tramadol | 22204-88-2 | 50 |
| Trihexyphenidyl | 144-11-6 | 0.1 |
| Trimethoprim | 738-70-5 | 0.1 |
| Venlafaxine | 93413-69-5 | 0.5 |
| Verapamil | 152-11-4 | 10 |
| Zolpidem | 99294-93-6 | 0.5 |

**Table S2.** Results for the optimization of extraction recoveries obtained with the D-optimal experimental design. The number of cycles varied between 1-3, the time varied between 5 and 20 and the formic acid addition varied between 0-5%. Only the extraction recoveries of the 4 active pharmaceutical substances that had lower extraction recoveries than 80% (as shown in Table 1) are included.

|  |  |  | **Extraction recoveries (%)** | | | |
| --- | --- | --- | --- | --- | --- | --- |
| **Number of cycles** | **Time (min)** | **% Formic Acid** | **Trimethoprim** | **Oxazepam** | **Flecainide** | **Amitriptilline** |
| 1 | 5 | 5 | 90 | 84 | 95 | 89 |
| 1 | 20 | 5 | 87 | 86 | 96 | 91 |
| 2 | 15 | 5 | 102 | 92 | 107 | 105 |
| 3 | 5 | 5 | 95 | 88 | 97 | 93 |
| 3 | 20 | 5 | 93 | 87 | 95 | 92 |
| 1 | 15 | 2.5 | 85 | 86 | 88 | 85 |
| 2 | 5 | 2.5 | 95 | 98 | 46 | 84 |
| 2 | 20 | 2.5 | 91 | 87 | 93 | 90 |
| 2 | 15 | 2.5 | 90 | 84 | 94 | 91 |
| 2 | 15 | 2.5 | 84 | 87 | 87 | 84 |
| 2 | 15 | 2.5 | 96 | 89 | 103 | 98 |
| 3 | 10 | 2.5 | 73 | 71 | 63 | 70 |
| 1 | 5 | 0 | 77 | 71 | 84 | 75 |
| 1 | 20 | 0 | 67 | 61 | 72 | 57 |
| 2 | 10 | 0 | 88 | 87 | 96 | 93 |
| 3 | 5 | 0 | 76 | 77 | 81 | 73 |
| 3 | 20 | 0 | 73 | 76 | 82 | 71 |

**Table S3.** Extraction recoveries for the active pharmaceutical substances tested for the three activated carbon adsorbents (ABC-W; AC; ABC-S). Each color for *Name* and *ATC code* represent a group of substances. The color coding of the extraction recovery represent the following: Red: <50% recovery; Yellow: <70% recovery; and Green: >70% recovery.

| **Name** | **ATC code** | **Function** | **ABC-W** | **AC** | **ABC-S** |
| --- | --- | --- | --- | --- | --- |
| Metoprolol | C07AB02 | Beta blocking agents, selective | 112 | 119 | 90 |
| Bisoprolol | C07AB07 |  | 111 | 111 | 92 |
| Eprosartan | C09CA02 | Angiotensin II receptor blockers (ARBs), plain | 84 | 109 | 94 |
| Irbesartan | C09CA04 |  | 56 | 59 | 59 |
| Telmisartan | C09CA07 |  | 41 | 24 | 20 |
| Atorvastatin | C10AA05 | HMG CoA reductase inhibitors | 2 | 1 | 5 |
| Rosuvastatin | C10AA07 |  | 60 | 36 | 50 |
| Oxytetracycline | J01AA06 | Tetracyclines | 59 | 42 | 26 |
| Tetracycline | J01AA07 |  | 53 | 52 | 45 |
| Roxithromycin | J01FA06 | Macrolides | 7 | 4 | 16 |
| Clarithromycin | J01FA09 |  | 8 | 6 | 15 |
| Azithromycin | J01FA10 |  | 21 | 17 | 25 |
| Ofloxacin | J01MA01 | Fluoroquinolones | 82 | 78 | 38 |
| Ciprofloxacin | J01MA02 |  | 36 | 24 | 15 |
| Norfloxacin | J01MA06 |  | 32 | 21 | 13 |
| Trihexyphenidyl | N04AA01 | Tertiary amines | 83 | 90 | 74 |
| Biperiden | N04AA02 |  | 85 | 87 | 75 |
| Fluphenazine | N05AB02 | Phenothiazines with piperazine structure | 14 | 3 | 9 |
| Perphenazine | N05AB03 |  | 0 | 1 | 4 |
| Flupentixol | N05AF01 | Thioxanthene derivatives | 31 | 22 | 26 |
| Chlorprothixene | N05AF03 |  | 34 | 23 | 17 |
| Clomipramine | N06AA04 | Non-selective monoamine re-uptake inhibitors | 67 | 66 | 58 |
| Amitriptyline | N06AA09 |  | 82 | 84 | 73 |
| Maprotiline | N06AA21 |  | 83 | 73 | 59 |
| Fluoxetine | N06AB03 | Selective serotonin re-uptake inhibitors | 90 | 66 | 59 |
| Citalopram | N06AB04 |  | 123 | 107 | 97 |
| Paroxetine | N06AB05 |  | 71 | 62 | 55 |
| Sertraline | N06AB06 |  | 66 | 72 | 59 |
| Mianserin | N06AX03 | Other antidepressants | 99 | 93 | 101 |
| Nefazodone | N06AX06 |  | 39 | 34 | 25 |
| Mirtazapine | N06AX11 |  | 93 | 101 | 78 |
| Bupropion | N06AX12 |  | 66 | 66 | 42 |
| Venlafaxine | N06AX16 |  | 114 | 111 | 94 |
| Duloxetine | N06AX21 |  | 55 | 38 | 19 |
| Diphenhydramin | R06AA02 | Aminoalkyl ethers | 97 | 96 | 80 |
| Clemastine | R06AA04 |  | 22 | 21 | 37 |
| Cyproheptadine | R06AX02 | Other antihistamines for systemic use | 129 | 104 | 103 |
| Fexofenadine | R06AX26 |  | 76 | 88 | 74 |
| Desloratadine | R06AX27 |  | 72 | 67 | 55 |
| Ranitidine | A02BA02 | [H2-receptor antagonists](https://www.whocc.no/atc_ddd_index/?code=A02BA) | 11 | 6 | 1 |
| dicycloverine | A03AA07 | [Synthetic anticholinergics, esters with tertiary amino group](https://www.whocc.no/atc_ddd_index/?code=A03AA) | 89 | 72 | 85 |
| loperamide | A07DA03 | [Antipropulsives](https://www.whocc.no/atc_ddd_index/?code=A07DA) | 77 | 62 | 69 |
| repaglinide | A10BX02 | [Other blood glucose lowering drugs, excl. insulins](https://www.whocc.no/atc_ddd_index/?code=A10BX) | 66 | 72 | 68 |
| dipyridamole | B01AC07 | [Platelet aggregation inhibitors excl. heparin](https://www.whocc.no/atc_ddd_index/?code=B01AC) | 32 | 17 | 44 |
| flecainide | C01BC04 | [Antiarrhythmics, class Ic](https://www.whocc.no/atc_ddd_index/?code=C01BC) | 88 | 94 | 73 |
| furosemide | C03CA01 | [Sulfonamides, plain](https://www.whocc.no/atc_ddd_index/?code=C03CA) | 8 | 11 | 11 |
| verapamil | C08DA01 | [Phenylalkylamine derivatives](https://www.whocc.no/atc_ddd_index/?code=C08DA) | 112 | 87 | 90 |
| diltiazem | C08DB01 | [Benzothiazepine derivatives](https://www.whocc.no/atc_ddd_index/?code=C08DB) | 113 | 89 | 87 |
| cilazapril | C09AA08 | [ACE inhibitors, plain](https://www.whocc.no/atc_ddd_index/?code=C09AA) | 133 | 129 | 126 |
| Finasteride | D11AX10 | [Other dermatologicals](https://www.whocc.no/atc_ddd_index/?code=D11AX) | 58 | 37 | 48 |
| bromocriptine | G02CB01 | [Prolactine inhibitors](https://www.whocc.no/atc_ddd_index/?code=G02CB) | 39 | 37 | 43 |
| alfuzosin | G04CA01 | [Alpha-adrenoreceptor antagonists](https://www.whocc.no/atc_ddd_index/?code=G04CA) | 82 | 66 | 40 |
| trimethoprim | J01EA01 | [Trimethoprim and derivatves](https://www.whocc.no/atc_ddd_index/?code=J01EA) | 76 | 92 | 63 |
| sulfamethoxazole | J01EE01 | [Combinations of sulfonamides and trimethoprim, incl. derivatives](https://www.whocc.no/atc_ddd_index/?code=J01EE) | 17 | 11 | 11 |
| clindamycin | J01FF01 | [Lincosamides](https://www.whocc.no/atc_ddd_index/?code=J01FF) | 85 | 61 | 6 |
| fluconazole | J02AC01 | [Triazole derivatives](https://www.whocc.no/atc_ddd_index/?code=J02AC) | 95 | 96 | 85 |
| flutamide | L02BB01 | [Anti-androgens](https://www.whocc.no/atc_ddd_index/?code=L02BB) | 40 | 31 | 37 |
| codeine | M01AE51 | [Propionic acid derivatives](https://www.whocc.no/atc_ddd_index/?code=M01AE) | 95 | 100 | 84 |
| Atracurium | ‎M03AC04 | [Other quaternary ammonium compounds](https://www.whocc.no/atc_ddd_index/?code=M03AC) | 27 | 38 | 22 |
| orphenadrine | M03BC01 | [Ethers, chemically close to antihistamines](https://www.whocc.no/atc_ddd_index/?code=M03BC) | 69 | 70 | 62 |
| buprenorphine | N02AE01 | [Oripavine derivatives](https://www.whocc.no/atc_ddd_index/?code=N02AE) | 69 | 33 | 63 |
| tramadol | N02AX02 | [Other opioids](https://www.whocc.no/atc_ddd_index/?code=N02AX) | 97 | 98 | 78 |
| dihydroergotamine | N02CA01 | [Ergot alkaloids](https://www.whocc.no/atc_ddd_index/?code=N02CA) | 43 | 23 | 33 |
| pizotifen | N02CX01 | [Other antimigraine preparations](https://www.whocc.no/atc_ddd_index/?code=N02CX) | 84 | 85 | 78 |
| clonazepam | N03AE01 | [Benzodiazepine derivatives](https://www.whocc.no/atc_ddd_index/?code=N03AE) | 97 | 47 | 69 |
| carbamazepine | N03AF01 | [Carboxamide derivatives](https://www.whocc.no/atc_ddd_index/?code=N03AF) | 86 | 103 | 89 |
| haloperidol | N05AD01 | [Butyrophenone derivatives](https://www.whocc.no/atc_ddd_index/?code=N05AD) | 90 | 64 | 46 |
| risperidone | N05AX08 | [Other antipsychotics](https://www.whocc.no/atc_ddd_index/?code=N05AX) | 62 | 61 | 29 |
| hydroxyzine | N05BB01 | [Diphenylmethane derivatives](https://www.whocc.no/atc_ddd_index/?code=N05BB) | 70 | 72 | 65 |
| flunitrazepam | N05CD03 | [Benzodiazepine derivatives](https://www.whocc.no/atc_ddd_index/?code=N05CD) | 76 | 48 | 82 |
| zolpidem | N05CF02 | [Benzodiazepine related drugs](https://www.whocc.no/atc_ddd_index/?code=N05CF) | 117 | 109 | 94 |
| caffeine | N06BC01 | [Xanthine derivatives](https://www.whocc.no/atc_ddd_index/?code=N06BC) | 103 | 76 | 87 |
| donepezil | N06DA02 | [Anticholinesterases](https://www.whocc.no/atc_ddd_index/?code=N06DA) | 92 | 67 | 64 |
| memantine | N06DX01 | [Other anti-dementia drugs](https://www.whocc.no/atc_ddd_index/?code=N06DX) | 114 | 97 | 96 |
| azelastine | R01AC03 | [Antiallergic agents, excl. corticosteroids](https://www.whocc.no/atc_ddd_index/?code=R01AC) | 115 | 82 | 77 |
| beclometasone | R01AD01 | [Corticosteroids](https://www.whocc.no/atc_ddd_index/?code=R01AD) | 51 | 33 | 50 |
| naloxone | V03AB15 | [Antidotes](https://www.whocc.no/atc_ddd_index/?code=V03AB) | 94 | 25 | 70 |

**Table S3.** Anatomical therapeutic chemical (ATC) classification codes for the first level. The second level consist of 2 digits explaining the therapeutic subgroup. The third level contains a letter and explains the therapeutical/pharmacological subgroup. The fourth level explains the chemical/therapeutical/pharmacological subgroup and consists of one letter. The fifth level indicates the chemical substance and consists of two digits. As an example, Atorvastatin has an ATC classification of C10AA05.

| Code (1st Level) | Content |
| --- | --- |
| A | Alimentary tract and metabolism |
| B | Blood and blood forming organs |
| C | Cardiovascular system |
| D | Dermatologicals |
| G | Genito urinary system and sex hormones |
| H | Systemic hormonal preparation, excluding sex hormones and insulins |
| J | Antiinfective for systemic use |
| L | Antineoplastic and immunomodulating agents |
| M | Musculo-skeletal system |
| N | Nervous system |
| P | Antiparasitic products, insecticides and repellents |
| R | Respiratory system |
| S | Sensory organs |
| V | Various |
